# Case report: Paralytic ileus resulted from nirmatrelvir/ritonavir-tacrolimus drug-drug interaction in a systemic lupus erythematosus patient with COVID-19

**DOI:** 10.3389/fphar.2024.1389187

**Published:** 2024-03-27

**Authors:** Wei Zhang, Xingguo Zhang, Jinming Han, Wen Zhang, Jiarui Xu, Xin Zhang, Hongjun Bian, Chong Meng, Deya Shang, Yi Zhou, Dawei Wang, Baobao Feng

**Affiliations:** ^1^ Department of Poisoning and Occupational Diseases, Shandong Provincial Hospital, Shandong University, Jinan, China; ^2^ Department of Emergency, Shandong Provincial Hospital Affiliated to Shandong First Medical University, Jinan, China; ^3^ Department of Poisoning and Occupational Diseases, Shandong Provincial Hospital Affiliated to Shandong First Medical University, Jinan, China; ^4^ Department of Pharmacy, Shandong Provincial Hospital Affiliated to Shandong First Medical University, Jinan, China

**Keywords:** paralytic ileus, nirmatrelvir-ritonavir, drug-drug interaction, systemic lupus erythematosus, COVID-19

## Abstract

Patients with systemic autoimmune rheumatic diseases are at a high risk for severe acute respiratory syndrome coronavirus 2 (SARS-CoV-2) infection and effective antiviral treatments including nirmatrelvir/ritonavir can improve their outcomes. However, there might be potential drug-drug interactions when these patients take nirmatrelvir/ritonavir together with immunosuppressants with a narrow therapeutic window, such as tacrolimus and cyclosporine. We present a case of paralytic ileus resulting from tacrolimus toxicity mediated by the use of nirmatrelvir/ritonavir in a patient with systemic lupus erythematosus (SLE). A 37-year-old female SLE patient was prescribed nirmatrelvir/ritonavir without discontinuing tacrolimus. She presented to the emergency room with symptoms of paralytic ileus including persistent abdominal pain, nausea, and vomiting, which were verified to be associated with tacrolimus toxicity. The blood concentration of tacrolimus was measured >30 ng/mL. Urgent medical intervention was initiated, while tacrolimus was withheld. The residual concentration was brought within the appropriate range and tacrolimus was resumed 8 days later. Physicians must be aware of the potential DDIs when prescribing nirmatrelvir/ritonavir, especially to those taking immunosuppresants like tacrolimus.

## 1 Introduction

Patients with systemic autoimmune rheumatic diseases (SARD) are at a high risk for severe acute respiratory syndrome coronavirus 2 (SARS-CoV-2) infection in terms of morbidity and mortality. Effective antiviral treatments including monoclonal antibodies, remdesivir, and oral medications such as nirmatrelvir/ritonavir and molnupiravir can improve the outcomes of SARD patients with COVID-19 (Qian et al., 2023). However, there might be potential drug-drug interactions (DDIs) since these patients take multiple medicines, including immunosuppressants with a narrow therapeutic window, such as tacrolimus and cyclosporine (Christians et al., 2002). Systemic lupus erythematosus (SLE) is a multi-system autoimmune disease with protean clinical manifestations. Paralytic ileus, also known as intestinal pseudo-obstruction, is a functional gastrointestinal disorder with symptoms of ileus, which may be idiopathic or secondary to other diseases as SARD, endocrine disorder, Parkinson’s disease and paraneoplastic syndrome. Tacrolimus, which has been widely used in the treatment of SLE, is metabolized in the liver and intestines by cytochrome P450 3A5 (CYP3A5) and 3A4 (CYP3A4) enzymes (Birdwell et al., 2015; Wang et al., 2023). The genetic polymorphisms of CYP genes, particularly CYP3A5, have a significant effect on tacrolimus pharmacokinetics, though the mechanism is still unclear (Wang et al., 2023). The ATP-binding cassette B subfamily 1 (ABCB1) encoding P-glycoprotein (P-gp) may also affect the metabolism of tacrolimus (Naito et al., 2015). Ritonavir is a CYP3A inhibitor and a P-gp inhibitor. It could increase the plasma concentration of medications that are substrates of CYP3A and P-gp enzymatic systems, such as tacrolimus, thus leading to toxic presentations (Sevrioukova and Poulos, 2010). Here we present a case of paralytic ileus resulting from tacrolimus toxicity mediated by the use of nirmatrelvir/ritonavir in a SLE patient with COVID-19.

## 2 Case description

A 37-year-old female presented to the emergency room with the chief complaint of persistent abdominal pain, nausea and vomiting at 1:00 a.m. on 8 January 2023. She had developed intermittent abdominal dull pain for 14 h and received no treatment. The patient was diagnosed with SLE with leukopenia 8 years ago, and thrombocytopenia 6 years ago. Her treatment regimen included: oral prednisone 10 mg/day, and oral tacrolimus 1 mg twice a day, which has been in place for 5 years. The blood concentration of tacrolimus was monitored regularly and her average tacrolimus trough level was 2.7 (target level of 2–6) ng/mL in the previous 6 months. She also took oral calcium tablets 600 mg/day, and oral vitamin D 1,000 U/day. She did not receive any vaccine against SARS-CoV-2. The patient developed a fever of 38.6°C, headache, and fatigue 4 days before admission, and was tested positive for SARS-CoV-2 antigen. Considering her immunocompromised and unvaccinated status, she was prescribed oral Paxlovid (nirmatrelvir/ritonavir) as: nirmatrelvir 300 mg combined with ritonavir 100 mg, twice a day, without adjustment of the immunosuppression regimen 3 days before her emergency visit.

**FIGURE 1:**
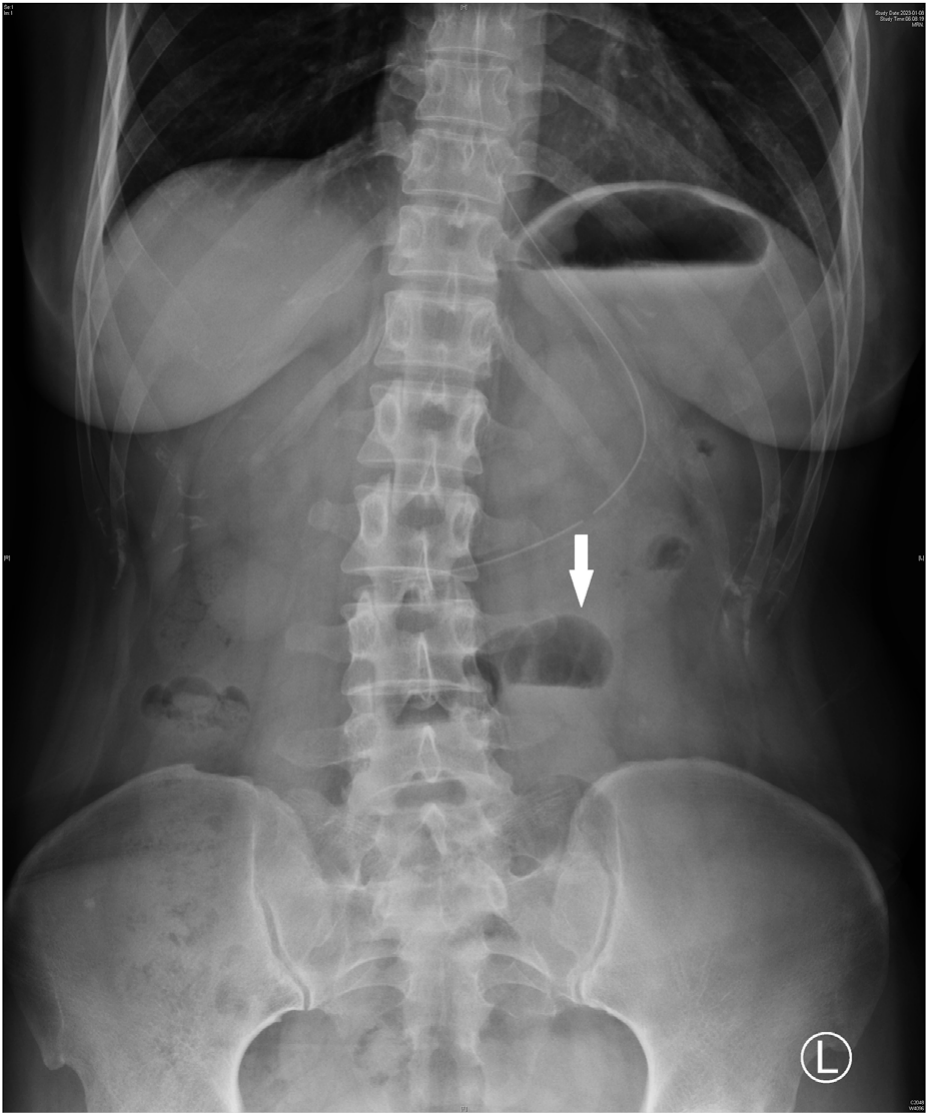
Abdominal X-ray imaging showed gas-liquid level in the mid-abdomen (white arrow).

**FIGURE 2:**
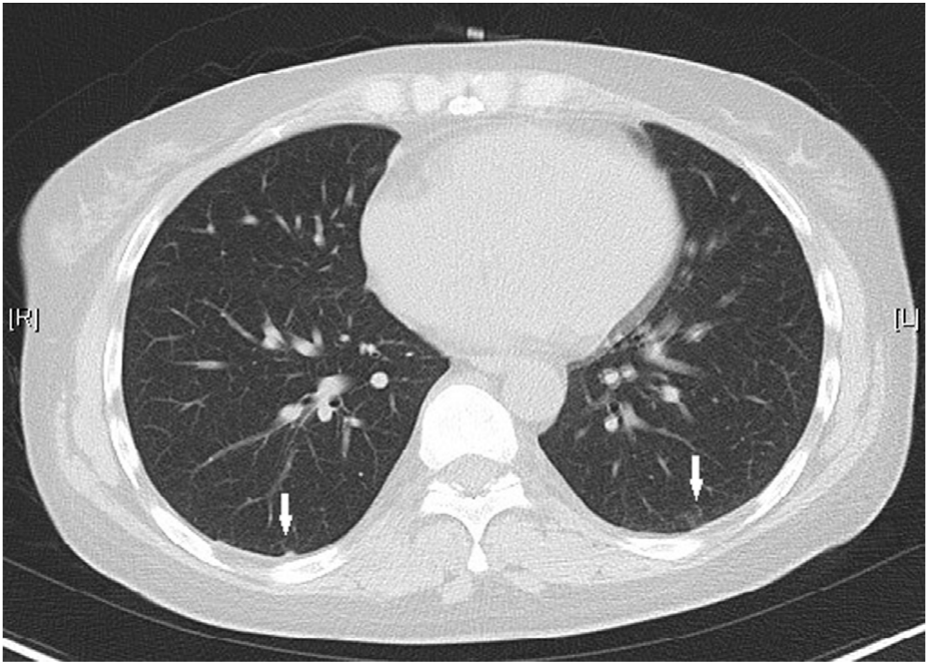
Chest CT showed small flaky high-density foci near the pleura on both sides (white arrows).

Upon admission, she was hemodynamically stable with a blood pressure of 100/67 mmHg, and an oxygen saturation of 99%. Abdominal tenderness and reduced bowel sounds were found through physical examination, while no other positive signs were noted. Laboratory examination revealed a slight hypocomplementemia (C3, 0.87 (reference range 0.9–1.8) g/L, C4, 0.18 (reference range 0.1–0.4) g/L), with a negative anti-dsDNA antibody, antiphospholipid-antibody and lupus anticoagulants. Blood cytometry was normal with erythrocyte of 4.3 × 10^12/L, leukocyte of 4.09 × 10^9/L, and platelet of 165 × 10^9/L. The serum potassium and sodium level were 3.64 mmol/L and 135.4 mmol/L respectively. Paralytic ileus was diagnosed by abdominal X-ray imaging (Figure 1) and was managed with diet ban, gastrointestinal decompression, enema, and parenteral feeding. Tacrolimus was withheld while nirmatrelvir/ritonavir was continued until 5 days of treatment were completed. The patient’s symptoms of abdominal pain and vomiting relieved, then diarrhea occurred on the second day. She was prescribed probiotics and the diarrhea improved 1 day later. The patient resumed a liquid diet on the third day. The tacrolimus trough blood concentration increased up to >30 ng/mL on the fifth day after the first nirmatrelvir/ritonavir dose (66 h after tacrolimus was stopped). Chest computed tomography (CT) took on the second day revealed bilateral small flaky high-density foci near the pleura (Figure 2), indicating mild pneumonia. These lesions disappeared when the patient took a second chest CT scan 3 months later. On the fifth day after admission, the blood concentration of tacrolimus decreased down to 17.20 ng/mL. The patient’s COVID-19 symptoms did not deteriorate. Her clinical condition improved and was discharged from hospital on the sixth day. During the hospitalization, no acute kidney injury was observed, with the serum creatinine level of around 0.70 mg/dL, and an estimated glomerular filtration rate of 110 mL/min/1.73 m^2^.

**FIGURE 3:**
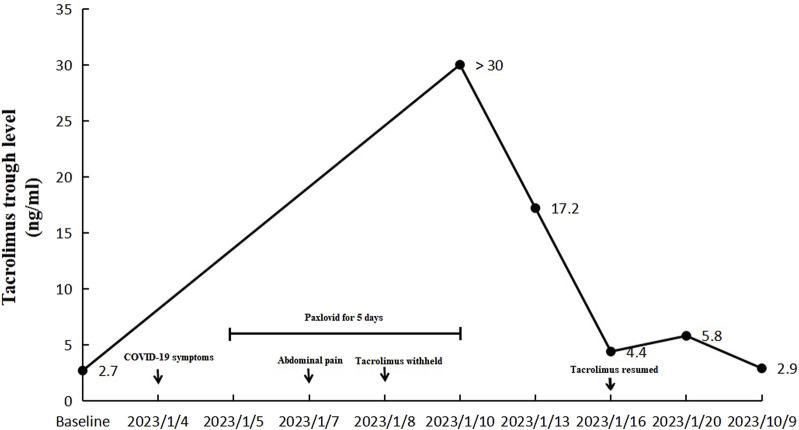
Changes of blood tacrolimus levels before, during and after the hospitalization (the dose of tacrolimus was 1 mg twice a day).

During follow-up, the blood concentration of tacrolimus decreased to 4.40 ng/mL on the eighth day after her emergency visit, and oral tacrolimus 1 mg twice a day was resumed. And the blood concentration of tacrolimus was 5.80 ng/mL 4 days after the re-initiation. The patient reported no symptoms of discomfort and continued the treatment regimen. Nine months later, the blood concentration of tacrolimus was 2.90 ng/mL (Figure 3).

## 3 Discussion

Nirmatrelvir/ritonavir is a medication authorized under emergency use by the US Food and Drug Administration for the treatment of mild to moderate COVID-19 infection in adults and children aged ≥12 years. Nirmatrelvir is a 3CL protease inhibitor of SARS-CoV-2. Ritonavir is a cytochrome P450 (CYP) 3A inhibitor and a P-glycoprotein (P-gp) inhibitor, which may increase the plasma concentration of medications that are substrates of CYP3A and P-gp enzymatic systems. Therefore, ritonavir has been used as a pharmacokinetic booster combined with other drugs such as anti-human immune deficiency virus drugs and direct acting antiviral drugs against hepatitis C virus (Lemaitre et al., 2023). *In vitro* studies showed that CYP3A4 is the major contributor (99%) to the oxidative metabolism of nirmatrelvir. However, the metabolism could be minimized when co-administered with ritonavir, resulting in higher plasma concentrations and longer half-life of nirmatrelvir (Lemaitre et al., 2023).

The combination of nirmatrelvir/ritonavir has significant and complex DDIs, mainly due to ritonavir. It is contraindicated to use nirmatrelvir/ritonavir with drugs that are highly dependent on CYP3A for clearance. Nirmatrelvir/ritonavir could also exhibit potential life-threatening interactions in individuals on immunosuppressants which are mainly metabolized by CYP3A. As one immunosuppressant metabolized by CYP3A, tacrolimus have been reported to have significant interactions with nirmatrelvir/ritonavir in some cases, most were from patients with solid organ transplant (Berar et al., 2022; Cadley et al., 2022; Kwon et al., 2022; Prikis and Cameron, 2022; Shah et al., 2022; Chen et al., 2023; Modi et al., 2023; Sindelar et al., 2023; Snee et al., 2023; Young et al., 2023; Zaarur et al., 2023). These patients presented with different symptoms including nausea, vomiting, fatigue, weakness, loss of appetite, abdominal pain, slowed speech, and peripheral neuropathy after they took nirmatrelvir/ritonavir without discontinuing tacrolimus. The blood concentrations of tacrolimus increased above the therapeutic threshold for all patients. And some were accompanied with acute kidney injury (Cadley et al., 2022; Kwon et al., 2022; Prikis and Cameron, 2022; Young et al., 2023).

CYP3A enzymes family are responsible for the oxidative metabolism of tacrolimus, among which only CYP3A4 and CYP3A5 are thought to be relevant in adults (Birdwell et al., 2015). The apparent clearance (CL/F) of tacrolimus is affected by genetic polymorphisms, particularly CYP3A5 genotype: patients with CYP3A5*1*1 and *1*3 genotypes had 39%–149% higher CL/F than those with CYP3A5*3*3 (CYP3A5 nonexpressers), indicating that they need to increase the starting dose 1.5 to 2 times to achieve the same blood concentrations as CYP3A5 nonexpressers (Wang et al., 2023). CYP3A4 has a lower effect on tacrolimus pharmacokinetics than CYP3A5. And CYP3A4*22 is thought to be the most significant polymorphic site that affects tacrolimus metabolism (Wang et al., 2023). So it was recommended to integrate both CYP3A5*3 and CYP3A4*22 genetic information in future development of tacrolimus population pharmacokinetics models to optimize initial dosing (Brunet et al., 2019). Tacrolimus is a substrate of P-gp, which is encoded by a member of the ATP-binding cassette B subfamily 1 (ABCB1). ABCB1 gene polymorphisms may also affect CL/F of tacrolimus (Naito et al., 2015). However, CYP3A4 and ABCB1 gene polymorphisms were not found to be significant factors for CL/F in a systematic review (Wang et al., 2023). The mechanism of gene polymorphisms on tacrolimus pharmacokinetics is still unclear, which needs to be furthur studied. Animal models showed that tacrolimus could accelerate gastric emptying, increase the frequency and amplitude of gastric contractions, resulted from its similarity with motilin, which induces contraction during the interdigestive period. In addition, tacrolimus also prolonged the intestinal transit time, resulting in gastrointestinal disorders such as reflux, diarrhea and constipation (Dall'Agnol and Hauschildt, 2014; Dall'Agnol et al., 2017).

Ritonavir is a type II ligand that perfectly fits into the CYP3A4 active site and irreversibly binds to the heme iron. The binding leads to decreased redox potential of the CYP protein, which is the basis of DDIs between ritonavir and immunosuppressant drugs such as tacrolimus, ciclosporin, everolimus and sirolimus that are metabolized by CYP3A (Sevrioukova and Poulos, 2010). A previous study showed that in the presence of steady-state concentrations of ritonavir (100 mg/day), the dose-normalized tacrolimus concentration at 24 h, and the area under the plasma concentration *versus* time curve from zero to infinity were 17-fold and 57-fold higher, respectively. The half-life of tacrolimus increased from 32 to 232 h (Badri et al., 2015). Keeping the same dose of tacrolimus following ritonavir treatment will lead to an extremely high tacrolimus exposure within 24 h. So, it is recommended to discontinue or reduce the dose of tacrolimus 12 h before nirmatrelvir/ritonavir therapy is started (Lemaitre et al., 2023). For those who continue to take tacrolimus, close therapeutic drug monitoring is needed. Devresse and others used a standard management strategy of tacrolimus dose adaptation (discontinuation of tacrolimus 12 h before the start of nirmatrelvir/ritonavir) for a case series of 14 kidney transplant recipients with COVID-19, no acute kidney injury was observed during the treatment course and tacrolimus was resumed 6 days later (Devresse et al., 2022). When patients present toxic symptoms and/or signs, tacrolimus must be discontinued and CYP inducers (phenytoin or rifampin) can be used to reverse the toxicity, which has been reported by some researchers (Cadley et al., 2022; Kwon et al., 2022; Shah et al., 2022; Sindelar et al., 2023; Snee et al., 2023; Xiong et al., 2023). However, the use of CYP inducers might compromise the effectiveness of nirmatrelvir/ritonavir treatment by increasing the metabolism of nirmatrelvir.

Based on previous researches, we recommend that tacrolimus be discontinued 12 h before initiation of nirmatrelvir/ritonavir, and resumed on day 7/8, either in the dose given prior to nirmatrelvir/ritonavir treatment or based on measured concentrations at the time of re-initiating tacrolimus treatment. Therapeutic drug monitoring of tacrolimus is not recommended during the 5-day treatment course of nirmatrelvir/ritonavir, since the risk of toxicity is limited when tacrolimus is discontinued before the antiviral treatment.

Tacrolimus has been widely used in the treatment of solid organ transplant patients, as well as individuals with SARD. It is recommended in the management of SLE (Fanouriakis et al., 2023). There have been studies on the safety and efficacy of nirmatrelvir/ritonavir in patients with SARD. The results showed a favorable outcome and acceptable safety profile of nirmatrelvir/ritonavir among a high-risk SARD population, and common adverse events reported were metallic taste, gastrointestinal upset and hypertension, which will not lead to drug discontinuation (Fragoulis et al., 2022; Gerolymatou et al., 2023). However, no detailed information about the DDIs between tacrolimus and nirmatrelvir/ritonavir in these patients were reported. SLE was reported to be associated with intestinal pseudo-obstruction, especially when the disease was active (Zhang et al., 2016; Wang et al., 2018). However, the SLE disease activity index of our case scored 2, while the SLE disease activity score was 1.12, both indicating remission of the disease. Paralytic ileus was also recognized as an extra-pulmonary manifestation of patients with severe COVID-19 (Ibrahim et al., 2020; Gulisano et al., 2023; Moghimi et al., 2023), which was not consistent with this case. Based on the above, the paralytic ileus was most likely result from the DDI between tacrolimus and nirmatrelvir/ritonavir in this report, supported by the toxicokinetic data.

Though there were cases of paralytic ileus reported to be associated with severe COVID-19 or active SLE, this is the first one inferred to be the result of DDI between tacrolimus and nirmatrelvir/ritonavir. This case provides another possible cause of paralytic ileus when dealing with patients with SLE and COVID-19, which enriches the literature.

There are limitations with this case report. Only experience of a single SLE patient was reported, which could not be generalized to the SARD population. A larger sample size is needed to establish generalizable findings. However, this would be difficult and time-consuming due to the relatively low incidence of paralytic ileus.

This case highlights the possible danger of outpatient nirmatrelvir/ritonavir prescriptions and the importance of risk mitigation strategies in minimizing DDIs. Since coronavirus continues to spread around the world, patients who are at high risk of developing severe COVID-19 must be treated with caution. Physicians must be aware of the potential DDIs when prescribing nirmatrelvir/ritonavir, especially to those taking immunosuppressants like tacrolimus. To avoid this, physicians must take detailed inquiries about the medical history and drug consumptions before prescribing nirmatrelvir/ritonavir. Medication instruction lists that clarifies necessary precautions, such as adverse drug reactions and DDIs, can be provided in outpatient settings. Effective patient education could help to ensure that patients are fully informed about the risks and benefits of the treatment they are going to receive. And patients should be encouraged to seek medical guidance and supervision when they have problems.

## Data Availability

The original contributions presented in the study are included in the article/supplementary material, further inquiries can be directed to the corresponding authors.
